# COVID-19 and vision impairment: Constraints negotiation, participation, and well-being during lockdown in the United Kingdom

**DOI:** 10.1177/02646196211009931

**Published:** 2023-01

**Authors:** Nigel Halpern, Jillian M Rickly, Marcus Hansen, John Fellenor

**Affiliations:** Kristiania University College, Norway; University of Nottingham, UK; Wrexham Glyndwr University, UK; Guide Dogs, UK

**Keywords:** Constraints Negotiation Theory, COVID-19, participation, vision impairment, well-being

## Abstract

In response to the Coronavirus disease, the United Kingdom (UK) government introduced lockdown measures requiring people to isolate and adhere to social distancing. This article uses Constraints Negotiation Theory to examine effects of the lockdown on people with vision impairment (PwVI). The research is based on an online survey of 639 PwVI in the UK. The analysis was conducted using partial least squares structural equation modelling in SmartPLS. The findings show that the lockdown had a negative effect on the participation and well-being of PwVI. However, they also show that the negative effects could be negotiated by adapting activities. This emphasises the need for a more inclusive response to current or future pandemics that recognises the vulnerabilities of PwVI and helps them to overcome the challenges associated with any measures that are introduced.

## Introduction

In March 2020, the World Health Organization (WHO) declared Coronavirus disease (COVID-19) a global pandemic. In response, the United Kingdom (UK) government introduced lockdown measures at the end of March 2020 (also known as a ‘stay-at-home order’). These required people to isolate, meaning they should only leave home for food, health reasons, or work (but only if unable to work from home). People were also required to adhere to social distancing, meaning if they did leave home, they should stay at least 2 m away from other people at all times. At the same time, the UK government identified ‘clinically vulnerable’ segments of the population who were urged to maintain the strictest forms of isolation and distancing and were able to access additional support (e.g. priority supermarket deliveries and other essential services).

People with vision impairment (PwVI) were not included as a clinically vulnerable population because vision impairment does not cause vulnerability to the virus. This was despite calls for a disability inclusive response to the crisis to prevent discrimination and health inequities and to maintain dignity (Armitage & Nellums, 2020). Vision impairment charities, activists, and academics argued that the aspects of daily life for PwVI do in fact increase their vulnerability to COVID-19 (Boyle et al., 2020; Crossland, 2020; Royal National Institute of Blind People [RNIB], 2020). For instance, PwVI often require closer or more tactile engagement with surfaces, objects, and people (e.g. to read Braille, hold objects closer to their face, use a magnifier or smartphone application to read labels, and seek assistance in shops and on public transport); the highly visual nature of distancing measures like the 2-m rule pose challenges for PwVI, as they cannot easily judge distance or see the 2-m marking barriers and signage, essentially limiting the agency of PwVI and countering the purpose of legislation that promotes equal opportunities and reduces social prejudices (Solomon et al., 2020); and there is evidence to suggest that PwVI are at greater risk from the effects of isolation (e.g. on loneliness) compared with the general population (Burholt et al., 2017; Hodge & Eccles, 2013). In April 2020, the British Broadcasting Corporation (BBC) began reporting a number of these challenges in their series ‘Coronavirus: Being Blind During the Pandemic’. Despite such efforts, government measures and their implementation failed to recognise the challenges faced by PwVI (Goggin & Ellis, 2020).

The vulnerabilities of PwVI, in association with the measures introduced, raised concerns about how the lockdown affected this group’s ability to maintain active, independent lives, and the subsequent impact that this has on well-being. In response, this article uses Constraints Negotiation Theory (CNT), which is introduced in the following sub-section, to examine effects of the lockdown on the participation and well-being of PwVI. In response to a call for data on the impacts of COVID-19 on people with disability (Reed et al., 2020), this article reports the findings from an online survey of PwVI in the UK. The survey was undertaken towards the end of the initial UK lockdown and as the first round of easing was occurring in some parts of the UK. A total of 639 complete responses were analysed using partial least squares structural equation modelling (PLS-SEM). Readers should note that in agreement with the research partner for this study, The Guide Dogs for the Blind Association (referred to hereafter as ‘Guide Dogs’), ‘vision impairment’ is used as the preferred terminology to ‘visual impairment’.

### Theory and hypotheses

CNT has an extensive history of theorisation, modelling, and construct development, especially within the field of leisure studies to understand factors affecting leisure participation and the extent to which they can be negotiated (Crawford et al., 1991; Hawkins et al., 1999; Jackson et al., 1993). A growing body of literature has expanded beyond the field of leisure studies including to disability studies (Burns & Graefe, 2007; Crawford & Stodolska, 2008; Henderson et al., 1995; Loucks-Atkinson & Mannell, 2007; Lyu et al., 2013; Ma & Ma, 2014; McKercher & Darcy, 2018; Park & Chowdhury, 2018).

In the literature, a constraint is generally considered to be any factor that acts as a perceived or actual barrier or hindrance to participation in an activity (Jackson et al., 1993). Traditionally, it has been argued that constraints are hierarchical and navigated sequentially (Crawford et al., 1991). Intrapersonal constraints, navigated first, relate to psychological states and are the most proximal to participation and most powerful to negotiate. Interpersonal constraints are navigated next and are based on social interactions and relationships. Structural constraints (e.g. time, finances, accessibility) are last to be navigated, most distant to participation, and least powerful to negotiate. However, some research suggests this might not always be the case. In particular, it has been observed among adults with cognitive disabilities that interpersonal constraints are much stronger as a result of complex caregiver relationships, less individual control over personal decision-making, and reduced agency in their resource management (Hawkins et al., 1999).

This study focuses on interpersonal constraints, which are relevant given that isolation and distancing measures aimed to reduce contact between people, and intrapersonal constraints, which are relevant given the potential impact of interpersonal constraints on people’s mental condition. Both constraints potentially affect participation directly. However, in line with CNT, they are expected to be hierarchical and navigated sequentially. In this study, interpersonal constraints are expected to have a negative effect on participation because of their positive effect on intrapersonal constraints (a mediating effect). Thus, the following hypotheses are proposed:

H1. Interpersonal constraints have a significant positive direct effect on intrapersonal constraints.
H2. Interpersonal constraints have a significant negative direct effect on participation.
H3. Intrapersonal constraints have a significant negative direct effect on participation.
H4. Intrapersonal constraints have a significant mediating effect on the relationship between interpersonal constraints and participation.

It is argued that participation is not dependent on the absence of constraints but on negotiation through them (Jackson et al., 1993; Loucks-Atkinson & Mannell, 2007; Lyu et al., 2013). Indeed, while the lockdown presented a number of constraints to participation, there were opportunities to negotiate them, and a key dimension to negotiating constraints rests in an individual’s ability to adapt, with or without support. For instance, all non-essential workers were expected to work from home during the lockdown, which requires some adaptation. Similarly, individuals were not supposed to be visiting friends or relatives from outside their own household, so they might adapt by interacting online. They might also shop for essential items online and have them delivered to their home instead of going shopping in person, and exercise at home or in less crowded areas to reduce the likelihood of coming into close contact with others. It means that under a higher level of adapting activities, the negative effect of interpersonal constraints on participation will be weaker, while under a lower level of adapting activities, the negative effect will be stronger (a moderating effect). Thus, the following hypotheses are proposed:

H5. Adapting activities has a significant positive direct effect on participation.
H6. Adapting activities has a significant moderating effect on the relationship between interpersonal constraints and participation.

It has been well-established in the disabilities literature that the ability to participate is essential for well-being (Beekman et al., 2002; Freedman et al., 2012; Schwanen & Ziegler, 2011). However, well-being has rarely been included in studies on constraints negotiation, despite the fact that it extends this body of literature and provides a more holistic picture of the role of participation and negotiation of constraints on quality of life (see Ma, 2008 for an exception). Well-being pertains to people’s subjective evaluations of their lives (Diener, 2009) and is relevant to this study, because in addition to being affected by participation, well-being may also be affected by intrapersonal constraints, for instance, due to concerns about how the virus may affect one’s own health. Indeed, the lockdown brought about immediate concerns regarding well-being for everyone in society, but particularly for those reliant on support in their daily lives (Son et al., 2021). Those that are able to negotiate constraints to maintain or increase participation are expected to have greater levels of well-being. In addition, intrapersonal constraints may have a negative effect on well-being because of their negative effect on participation (a mediating effect). Hence, the following hypotheses are proposed:

H7. Intrapersonal constraints have a significant negative direct effect on well-being.
H8. Participation has a significant positive direct effect on well-being.
H9. Participation has a significant mediating effect on the relationship between intrapersonal constraints and well-being.

**Figure 1.**
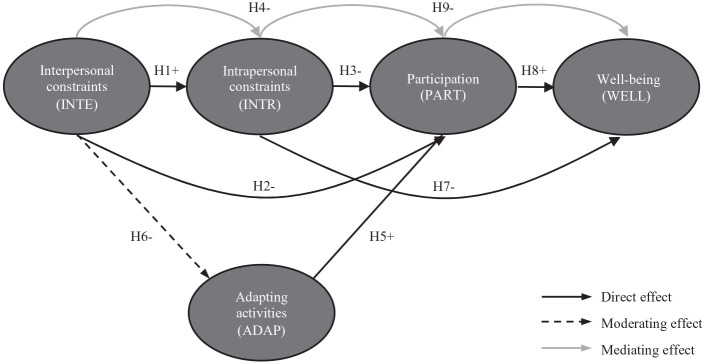
Theoretical model with numbered hypotheses (H) and expected direction (+ for positive, − for negative).

The theoretical model for this study is illustrated in Figure 1.

The ability to negotiate constraints has been conceptually related to the ‘hierarchy of social privilege’ from its earliest development (Crawford et al., 1991). This was originally related to social class, with the assumption that income and education have an indirect effect on the perception and experience of constraints, and subsequently affect participation. However, this has been investigated further among adults with cognitive impairment, finding direct effects influenced by social relationships and society, as opposed to factors associated with higher social privilege (Hawkins et al., 1999). The additional literature presents a more nuanced picture, suggesting varying degrees of constraint and participation, for instance, based on gender (Henderson et al., 1995). As such, seven conditions or ‘respondent characteristics’ are included in this study as control variables: gender, age, income, household composition, severity of vision impairment, guide dog ownership, and underlying health problems specific to COVID-19. This study examines the effect of these conditions on participation as an outcome of the constraint’s negotiation process.

## Method

The survey was developed in collaboration with Guide Dogs, a charity that supports PwVI in the UK by providing guide dogs, mobility, and other rehabilitation services. Ethical approval was granted by Nottingham University Business School Research Ethics Committee on 29 April 2020. Key constructs were needed for the analysis and are described as follows (see also Table 1 for specific wordings of the questions and items used to create each construct).

**Table 1.** Survey items for key constructs.

Label	Items
Compared to usual [the COVID-19 lockdown means that], I have been:	
INTE1	More concerned about coming into contact with others
INTE2	More frustrated by others not behaving as they should
INTE3	More concerned about the well-being of my loved ones
INTE4	More concerned about how my actions might affect the health of others
Compared to usual [the COVID-19 lockdown means that], I have been:	
INTR1	Less motivated to do things in daily life
INTR2	More concerned for my own health
INTR3	More worried about everything
INTR4	More confused about what I should be doing
How active have you been [during the COVID-19 lockdown compared to usual] in terms of:	
PART1	Physical independence (e.g. housework, cooking, self-care)
PART2	Keeping in touch with others (e.g. with friends and family)
PART3	Exercise, hobbies, or other leisure activities
PART4	Work, study, or regular volunteering
PART5	Mobility (e.g. using public transport to get around)
Compared to usual [during the COVID-19 lockdown]:	
WELL1	I have been in a good state of mind (e.g. in terms of happiness, anxiety, loneliness)
WELL2	I have been satisfied with my life overall
WELL3	I have felt optimistic about the future
WELL4	My sleep has been restless^a^

Response scales: ‘PART’ items: 1 = *much less active*; 2 = *slightly less active*; 3 = *about the same*; 4 = *slightly more active*; 5 = *much more active*.

All others: 1 = *strongly disagree*; 2 = *tend to disagree*; 3 = *neither disagree nor agree*; 4 = *tend to agree*; 5 = *strongly agree*.

a Reversed for the analysis.

### Interpersonal constraints (INTE)

In CNT, these are typically associated with social interactions and personal relationships. In the context of this study, they are likely to be associated with the need to isolate and comply with distancing measures. Therefore, items were included regarding concern about contact with others (INTE1); frustration with the behaviour of others (INTE2); concern about the well-being of loved ones (INTE3); and concern about how one’s own actions may affect others (INTE4).

### Intrapersonal constraints (INTR)

In CNT, these are typically associated with psychological attributes that interact with activity preferences, therefore acting as determinants of (dis)interest in participation. In this study, the focus is on psychological states related to reduced motivation (INTR1); concern for one’s own health (INTR2); increased worry (INTR3); and possible confusion about what one should be doing (INTR4).

### Participation (PART)

According to the WHO (2001), participation refers to a person’s involvement in a life situation such as employment, education, or relationships. Items were used in this study to measure five categories of participation: physical independence (PART1); keeping in touch with others (PART2); exercise, hobbies or other leisure activities (PART3); work, study or regular volunteering (PART4); and mobility (PART5). These five categories appear in multiple disability studies on participation (Perenboom & Chorus, 2003).

### Negotiation (ADAP)

The lockdown aimed to reduce ‘normal’ approaches to participation for most of the population. However, negotiation of constraints through adapting activities is expected to be central to maintaining participation. It means that constraints are not simply barriers, but also opportunities for thinking differently. Adapted activities were measured using a single item: ‘[During the COVID-19 lockdown] I have been adapting my regular activities so that I can keep doing them’.

### Well-being (WELL)

As a broad concept, well-being can be measured using a diverse range of subjective items, for instance, related to a person’s state of mind, health, resilience, efficacy, relationships, and access to resources. This study used items adapted from previous studies such as Huppert et al. (2009) that measure well-being according to overall state of mind (WELL1), satisfaction with life (WELL2), optimism about the future (WELL3), and quality of sleep (WELL4).

### Control variables

Respondent characteristics were included as control variables (coded 1 for *Yes* and 0 for *No*). The variables were female gender (FEM), aged 70+ (70+), household income of less than £25,000 (INC), live alone (LIV), severe vision impairment (SEV), guide dog owner (GDO), and underlying health problems specific to COVID-19 (UHP).

Online survey platform Qualtrics was used for the survey. An initial version of the survey was created using question formats deemed as being accessible to respondents who use third-party screen readers, as is common for PwVI. This was tested by Guide Dogs and resulted in suggestions to improve accessibility. After implementing these, a pilot survey was conducted with four PwVI. Feedback highlighted challenges associated with questions in profile matrix format when using some screen readers, which added significantly to the time and effort needed to complete the survey. As a result, profile matrices were replaced by multiple-choice questions to ensure access via all screen readers.

An invitation for PwVI to participate in the survey, including a link to it, was emailed by Guide Dogs to their members on 19 May 2020 and to a list of carers on 22 May 2020. Also, on 22 May 2020, Visionary – an organisation that represents sight loss charities in the UK – sent the survey invitation by email to their members. Recipients were given an option to complete the survey by telephone and 16 people chose this option. The survey closed on 7 June 2020 at which time 937 complete responses had been received. As PLS-SEM is used for the analysis to investigate relationships between key constructs, only those that provided valid responses to all the items in Table 1 were included in the analysis. This provided a final sample of 639 responses. Those without valid responses to sample characteristics were still included in the analysis, meaning that *N* varied for those variables (Table 2), and mean replacement was used for missing values in the analysis.

**Table 2.** Sample characteristics.

Characteristic	Category	*N*	Respondents (valid %)
Gender	Female	394	62.5
	Male	236	37.5
Age	18–29	45	7.1
	30–39	67	10.6
	40–49	89	14.1
	50–59	174	27.6
	60–69	165	26.2
	70+	90	14.3
Household income	Less than £25,000	264	53.4
	£25,001–£50,000	165	33.4
	More than £50,000	65	13.2
Household composition^a^	Live alone	169	24.9
	Live with one or more adults	441	64.9
	Live with one or more children	69	10.2
Severity of vision impairment	Mild	98	17.8
	Moderate	113	20.5
	Severe	340	61.7
Guide dog owner	Yes	225	37.3
	No	378	62.7
Underlying health problems	Yes	221	34.6
	No	418	65.4

a Respondents could select more than one response.

## Results

### Descriptive results

Descriptive statistics for each item are listed in Table 3. In terms of interpersonal constraints, respondents were particularly concerned for their loved ones (INTE3, *M* = 4.5). Responses to items measuring intrapersonal constraints were lower than those that measure interpersonal constraints and the higher standard deviations, especially for INTR4, show that individual responses were less clustered around the mean. On average, respondents *tended to agree* about being more concerned for their own health (INTR2, *M* = 3.7) and more worried about everything (INTR3, *M* = 3.5). However, they *neither agreed nor disagreed* about being less motivated to do things in daily life (INTR1, *M* = 3.2) and about feeling more confused by what they should be doing (INTR4, *M* = 2.8).

**Table 3.** Descriptive statistics for each item.

Label	% respondents					*M*	*SD*
	1	2	3	4	5		
INTE1	4.4	5.0	6.1	32.2	52.3	4.2	1.062
INTE2	5.0	5.9	9.1	40.4	39.6	4.3	1.020
INTE3	4.1	3.0	8.6	30.2	54.1	4.5	0.813
INTE4	2.0	1.6	3.6	28.0	64.8	4.0	1.084
INTR1	14.9	20.0	16.1	32.1	16.9	3.2	1.330
INTR2	6.7	11.0	16.1	37.9	28.3	3.7	1.184
INTR3	10.0	13.8	17.5	37.1	21.6	3.5	1.249
INTR4	24.5	22.5	16.4	25.2	11.5	2.8	1.362
PART1	26.9	23.6	30.5	9.7	9.2	2.5	1.241
PART2	30.5	16.9	21.8	18.6	12.2	2.7	1.395
PART3	46.9	14.4	24.3	8.1	6.3	2.4	1.400
PART4	39.9	20.8	15.5	12.4	11.4	2.1	1.260
PART5	82.8	7.3	7.7	1.5	0.7	1.3	0.731
ADAP	11.0	20.3	19.9	36.9	11.9	3.2	1.206
WELL1	14.7	24.9	21.4	25.8	13.1	3.0	1.274
WELL2	21.6	30.2	17.2	18.0	13.0	3.0	1.207
WELL3	12.4	23.8	21.6	32.2	10.0	2.7	1.160
WELL4	16.6	28.6	26.6	21.9	6.3	2.7	1.335

‘PART’ responses: 1 = *much less active*; 2 = *slightly less active*; 3 = *about the same*; 4 = *slightly more active*; 5 = *much more active*.

All other responses: 1 = *strongly disagree*; 2 = *tend to disagree*; 3 = *neither disagree nor agree*; 4 = *tend to agree;* 5 = *strongly agree*.

In terms of participation, mobility was most affected during lockdown with respondents being *much less active* compared with before it (PART5, *M* = 1.3). The low *SD* of 0.731 shows that reduced mobility was widespread among the sample. Indeed, 83% of respondents were *much less active*. A further 7% were *slightly less active*. The second most affected type of participation was work, study, and volunteering (PART4, *M* = 2.1), and this is followed by exercise, hobbies, and other leisure activities (PART3, *M* = 2.4). However, activity levels stayed *about the same* for physical independence (PART1, *M* = 2.5) and keeping in touch with others (PART2, *M* = 2.7). Respondents *neither agreed nor disagreed* that they adapted activities during lockdown (ADAP, *M* = 3.2) although there was some degree of variation in responses with an *SD* of 1.206. Indeed, 49% of respondents *agreed*, while 31% *disagreed*, and 20% *neither agreed nor disagreed*.

With regard to well-being, quality of sleep (WELL4) and optimism about the future (WELL3) both had the lowest *M* score of 2.7, while state of mind (WELL1) and overall satisfaction with life (WELL2) both had the highest *M* score of 3.0. There was a fair degree of variation in responses for individual items and therefore people’s well-being. For instance, 52% of respondents disagreed about being satisfied with their life overall (WELL2), 31% *agreed*, and 17% *neither agreed nor disagreed*.

### Measurement model results

**Figure 2.**
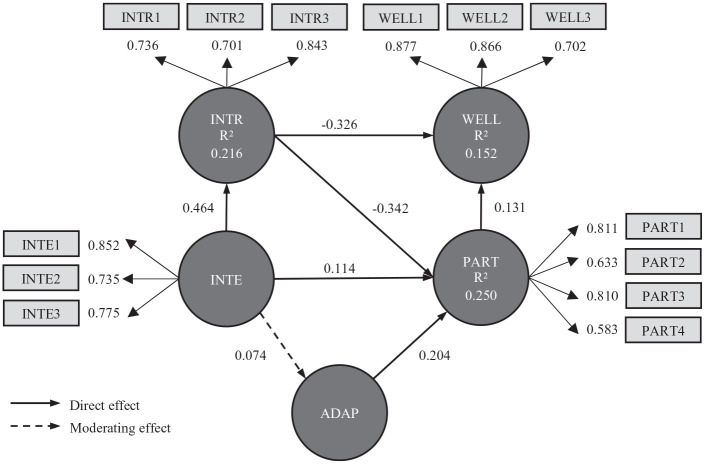
Structural model with item loadings, path coefficients for direct and moderating effects, and *R*^2^ for endogenous constructs.

A reflective model was used to test the hypotheses (Figure 2). Several steps are recommended to assess reflective models created using SEM-PLS (Hair et al., 2019). The first is to examine the loading values of individual items, which should exceed 0.7. Loadings of 0.4–0.7 can be retained if convergent validity is achieved with a recommended average variance explained (AVE) of more than 0.5 (Hair et al., 2014). Three items in Table 3 (INTE4, INTR4, and PART5) were removed despite having loadings of 0.4–0.7, because doing so improved AVE. As can be seen in Figure 2, PART2 and PART4 have loadings below 0.7. Removing them did not improve AVE so they were retained. The next step was to examine internal consistency reliability. Composite reliability (CR) is recommended where a value of 0.6–0.9 is considered satisfactory (Hair et al., 2019). Thresholds for AVE and CR are met (Table 4). Cronbach’s alpha (α) can also be used and assumes similar thresholds to CR. A third step was to assess discriminant validity, which is the extent to which latent constructs are distinct from one another (Hair et al., 2017). The heterotrait–monotrait (HTMT) ratio is generally considered to be the best approach (Henseler et al., 2015). Values of less than 0.85 are recommended (Hair et al., 2019). All of the values in this study were 0.68 or below (Table 4) meaning discriminant validity is accepted.

**Table 4.** Reliability, validity, and collinearity statistics.

	Reliability and validity^a^			Discriminant validity (HTMT)^b^				Collinearity (VIF)^c^		
	α	CR	AVE	INTR	INTE	PART	WELL	INTR	PART	WELL
INTR	.639	0.806	0.582	–	–	–	–	–	1.369	1.120
INTE	.705	0.831	0.623	0.681	–	–	–	1.000	1.316	–
PART	.679	0.805	0.514	0.478	0.083	–	–	–	–	1.120
WELL	.761	0.858	0.671	0.478	0.185	0.312	–	–	–	–
ADAP	–	–	–	–	–	–	–	–	1.031	–

a Figures for key constructs only.

b Figures compare latent constructs only.

c Figures for inner model paths only.

The structural model was then estimated using the PLS algorithm in SmartPLS. Collinearity was examined to ensure that it does not bias the regression results. This was assessed using variance inflation factors (VIFs) for inner model paths, which should have values of less than 5, although collinearity can also occur at lower values of 3–5 (Hair et al., 2019). Inner VIF values ranged from 1.00 to 1.37 (Table 4), meaning collinearity was not a problem. Regarding model fit, the standardised root mean residual (SRMR) of 0.065 is within the recommended threshold of 0.08 (Henseler et al., 2016).

### Structural model results

The significance of path coefficients and *f*^2^ effect sizes (an alternative to path coefficients that show how the removal of a predictor construct affects an endogenous construct’s *R*^2^ value) was determined using Bootstrapping with 5000 bootstrap re-samples (Table 5). H1–H9 are accepted, although H2 has a positive effect (a negative effect was expected). The result means that the more concerned PwVI have been about social interactions and relationships, the more active they have been during the lockdown. There is anecdotal evidence of this for the population more generally, for instance, with people doing more chores at home, including gardening and home maintenance. Well-being of loved ones is an item of interpersonal constraints for which increased concern may mean people, especially those that adapted (e.g. using telephone or online communications), have had more contact than normal with loved ones during the lockdown. Similarly, there may have been increased levels of productivity of people working or studying from home instead of needing to commute to/from work or study, and from attending meetings or classes online versus attending in person. People have also been keen to get their daily exercise in, or to be more active with their hobbies or leisure activities or take up new ones. Some have done more shopping to stockpile certain items such as toilet roll and cupboard staples.

**Table 5.** Path coefficients, effect sizes, and hypothesis result.

Path	Coefficient	Effect (*f*^2^)^a^	*SD*	*t* statistic	*p* value	Hypothesis result
Direct effects						
INTE-INTR	0.464	0.280	0.041	11.352	.000	H1 accepted
INTE-PART	0.114	0.013	0.037	3.064	.002	H2 accepted^b^
INTR-PART	−0.342	0.110	0.041	7.970	.000	H3 accepted
ADAP-PART	0.204	0.050	0.038	5.427	.000	H5 accepted
INTR-WELL	−0.326	0.110	0.041	7.963	.000	H7 accepted
PART-WELL	0.131	0.020	0.041	3.220	.001	H8 accepted
Mediating effects						
INTE-INTR-PART	−0.159	–	0.024	6.714	.000	H4 accepted
INTE-PART-WELL	0.015	–	0.007	2.213	.027	Not hypothesised
INTR-PART-WELL	−0.045	–	0.015	2.975	.003	H9 accepted
INTE-INTR-WELL	−0.152	–	0.021	7.121	.000	Not hypothesised
ADAP-PART-WELL	0.027	–	0.010	2.603	.009	Not hypothesised
INTE-INTR-PART-WELL	−0.021	–	0.007	2.808	.005	Not hypothesised
Moderating effect						
INTE-ADAP-PART	0.074	–	0.032	2.292	.022	H6 accepted
Control variables						
FEM-PART	0.062	0.01	0.036	1.747	.081	Positive effect^c^
70+-PART	−0.067	0.01	0.037	1.828	.068	Negative effect^c^
INC-PART	−0.113	0.02	0.038	2.946	.003	Negative effect
LIV-PART	−0.038	0.00	0.035	1.078	.281	No effect
SEV-PART	−0.081	0.01	0.040	2.030	.043	Negative effect
GDO-PART	−0.130	0.02	0.039	3.370	.001	Negative effect
UHP-PART	−0.124	0.02	0.037	3.398	.001	Negative effect

a *f*^2^ thresholds for significant direct effects: 0.02 = *weak*, 0.15 = *moderate*, 0.35 = *strong* (Cohen, 1988).

b Significant effect is accepted but the direction is positive when the hypothesis expected it to be negative.

c Significant, but only at the 10% level of confidence. All other noted effects are significant at the 5% level of confidence.

Interpersonal constraints had a moderate positive effect on intrapersonal constraints (H1). Intrapersonal constraints subsequently affected participation (H3) and well-being (H7). Participation also affected well-being (H8) and was affected by one’s ability to adapt (H5). While intrapersonal constraints affected participation, the effect of interpersonal constraints (H2) falls short of the threshold for a weak effect (0.01). Interestingly though, the relationship between interpersonal constraints and participation is fully mediated by intrapersonal constraints (H4), meaning that interpersonal constraints (through intrapersonal constraints) affected participation. The other indirect effect hypothesised is the mediating effect of participation on the relationship between intrapersonal constraints and well-being (H9). This effect is significant but weak (−0.045, *p* = .003).

One indirect effect that was not hypothesised but is worth mentioning is the mediating effect of intrapersonal constraints on the relationship between interpersonal constraints and well-being (0.152, *p =* .000), meaning that interpersonal constraints through intrapersonal constraints affected well-being. Although not shown in Figure 2, the path INTE-WELL was checked and found to have a path coefficient of 0.033 (*p =* .504). As the direct effect was not significant but the indirect effect was, it can be concluded that intrapersonal constraints had a full mediating effect on the relationship between interpersonal constraints and well-being.

**Figure 3.**
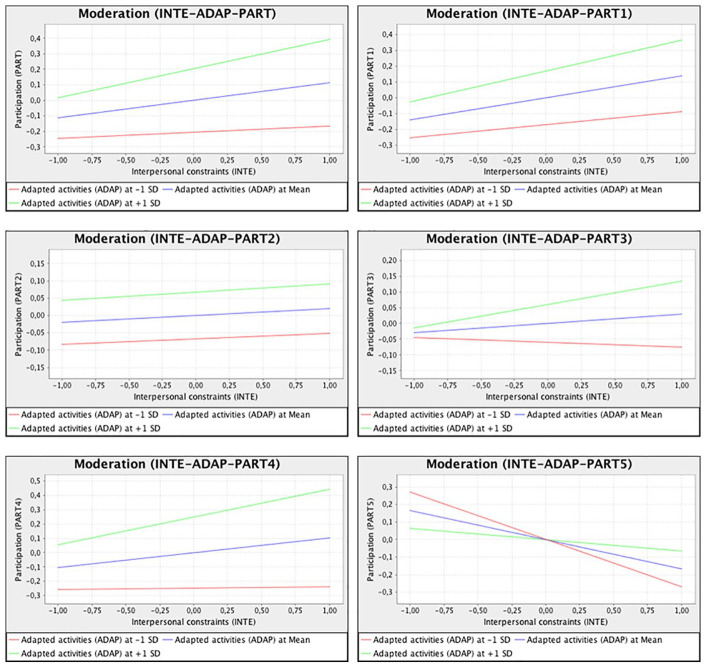
Moderating effect of adapted activities on the relationship between interpersonal constraints and participation.

The moderation effect of adapted activities on interpersonal constraints and participation (H6) is illustrated in Figure 3. The red line shows the relationship between interpersonal constraints and participation when adapted activities was lower (with a value of 1 below the *SD*). It shows that as interpersonal constraints increased, so did participation, but only slightly. The blue line represents an average level of adapted activities, while the green line represents a higher level (with a value of 1 above the *SD*). Under a higher level of adapted activities, the positive relationship between interpersonal constraints and participation was much stronger.

The mobility item (PART5) was excluded from the participation construct because it had a weak loading. Figure 3 shows the moderating effect of adapted activities on the relationship between interpersonal constraints and each individual component of participation (as well as the excluded mobility item PART5), where it can be seen that increased interpersonal constraints (at mean levels of adapted activities) resulted in increased participation for all but PART5. Greater levels of adapted activities strengthened the relationships in a positive way (although the rate of increase for PART2 remains about the same), except for with PART5, which had a weaker negative relationship. Mobility has therefore been negatively affected and more difficult to overcome through adapted activities during lockdown compared with other components of participation.

Regarding the control variables (Table 5), all but one of them (live alone, LIV) were found to have a significant direct effect on participation at the 10% level (*p* < .10). However, household income of less than £25,000 (INC), guide dog ownership (GDO), and underlying health problems specific to COVID-19 (UHP) were the only ones to meet the stricter 5% level of significance (*p* < .05) and the *f*^2^ threshold of 0.02 – all of the effects being negative.

## Discussion

In support of previous studies (Jackson et al., 1993; Loucks-Atkinson & Mannell, 2007; Lyu et al., 2013), this study finds that participation is enhanced by the ability to negotiate constraints. In particular, it finds that adapting activities to reduce and overcome interpersonal constraints associated with isolation and distancing increases participation among PwVI. There is anecdotal evidence of people being more active during the lockdown as a result of adapting their activities, and the findings of this study provide empirical support for this among PwVI. One exception is with mobility, which was not included as a component of participation but is shown, post hoc, to have been more difficult for PwVI to overcome through adaptation during the lockdown.

The findings emphasise the importance of support and intervention strategies that allow PwVI to adapt their daily activities to the lockdown to avoid reduced levels of participation and well-being. Technological solutions may feature heavily here, especially those that can help PwVI to navigate and comply with distancing measures, but also to reduce contact with surfaces that may carry the virus. For instance, smartphone applications can potentially assist with navigation or can connect to other devices via Bluetooth or the internet to facilitate touchless solutions (e.g. for mobile payments or to access information via scannable QR codes). Smartphone applications but also other solutions such as sonar equipped smart canes also have the possibility of alerting PwVI (e.g. via vibrations) if they get within a certain distance of another person or object, therefore helping to support distancing measures.

Governments and/or charities should also assess the need for campaigns to increase awareness among service providers and the general public of the challenges faced by PwVI during COVID-19, and how to assist them to overcome those challenges as the pandemic continues. Similarly, campaigns might also focus on equipping PwVI with the skills needed to adapt to a world where distancing measures and touchless services might be the new normal.

Well-being is scarcely covered in the CNT literature. In the findings of this study, well-being was negatively affected by intrapersonal constraints and positively affected by participation, emphasising the impact that psychological state and the ability to lead independent and active lives had on the well-being of PwVI during the lockdown. The findings support literature on the importance of participation for well-being among people with disabilities (Beekman et al., 2002; Freedman et al., 2012; Schwanen & Ziegler, 2011), and on the negative effect that COVID-19 has had on the participation and well-being of vulnerable populations (Son et al., 2021). This further emphasises the need for support and intervention strategies that allow PwVI to adapt. However, governments and/or charities need to assess whether mental health services are sufficient enough for PwVI who are less able to adapt and whose well-being may be affected as a result.

The findings also contribute to theory on the hierarchy of social privilege (Crawford et al., 1991), demonstrating the effect of income on participation. This is a particular concern given the high proportion of PwVI that have a low household income. For instance, in a survey of guide dog owners in the UK, 60% of respondents reported a total annual household income of £25,000 or less (Rickly et al., 2019). The figure is 53% for respondents to this survey, which includes PwVI that do not own a guide dog.

The findings also recognise the effect of other conditions that are either specific to PwVI or to the pandemic and have therefore not been considered in previous CNT studies. In particular, PwVI that own a guide dog or have underlying health problems specific to COVID-19 experienced significantly lower levels of participation during the lockdown. The finding regarding guide dog ownership is interesting and warrants further investigation, because it is not immediately clear why PwVI that own a guide dog would have significantly lower levels of participation. One explanation could be that owners are concerned about their dog’s lack of training to deal with distancing measures and are therefore less confident to venture out with their dog. This would have significant implications for the training of guide dogs (or encouragement to use alternative aids such as a cane) as a mechanism for enabling PwVI to better negotiate constraints to participation. An additional or alternative explanation could be that PwVI who have a guide dog are generally more active than PwVI who do not. Guide dog owners who consider themselves to be quite active normally might therefore have felt much less active during the lockdown. This builds on the already known benefits of guide dogs to PwVI, for instance, on physical independence and mobility (Audrestch et al., 2015).

Regarding underlying health problems, vision impairment is often comorbid (McLean et al., 2014). For instance, in a survey of guide dog owners in the UK, 41% of respondents claimed to have an additional disability or medical condition to vision impairment (Rickly et al., 2019), while in this survey, 35% of respondents have underlying health problems that make them specifically vulnerable to COVID-19. Comorbidity has been shown to increase the risk of COVID-19 infection (Boyle et al., 2020), and the high prevalence of comorbidities specific to COVID-19 among PwVI means that they would be expected to be isolating for longer than the general population. People are negatively affected when experiencing isolation or perceived social isolation (i.e. experiencing reduced cognitive performance, accelerated cognitive decline and depression) (Andersson et al., 2015; Cacioppo & Hawkley, 2009), and there is evidence to suggest that PwVI are at greater risk from the effects of isolation compared with the general population (Hodge & Eccles, 2013). Disability in general has a significant indirect effect on loneliness (Burholt et al., 2017), further highlighting the risks associated with isolation and distancing for PwVI. In addition, evidence suggests that increased levels of stress, shifts in nutrition patterns and reduced access to essential services (e.g. resulting from isolation and distancing) can potentially interact with, and exacerbate, a range of disabilities or medical conditions (Kalantar-Zadeh & Moore, 2020). As discussed earlier in the context of well-being, this emphasises the need to assess whether mental health services and health services more generally are sufficient enough for PwVI who may be more prone to loneliness or other health effects associated with isolation or distancing. It also calls for those with comorbidity to be prioritised for early access to a COVID-19 vaccine.

A limitation of this study is that it only surveys PwVI, so a comparison cannot be made of the impact the lockdown has had on PwVI compared with people with other disabilities, or with the population in general. This would be an area of interest for further research. Also, the findings are limited to the UK. It would be interesting to compare lockdown effects on PwVI in other countries where lockdown measures but also support and intervention strategies for PwVI might have varied.

The survey for this study took place just as the UK was beginning to ease its initial lockdown measures, therefore representing the opinions of PwVI at a specific period-in-time. It would be worthwhile to conduct follow-up studies to investigate the effects of ongoing measures and also to investigate more long-term effects of the pandemic, including how the introduction and withdrawal of different measures impact on PwVI.

It is arguably a normal human response to experience increased interpersonal constraints during a lockdown, as limiting people’s movements and contact with others is aimed at reducing the spread of the virus. Some people will be better than others at negotiating constraints to cope with the situation. However, there will be a point at which there are more serious repercussions for one’s psychological state and overall well-being, and additional support and intervention will be needed to reduce the likelihood of people reaching that threshold. In terms of further research, it would be worthwhile to investigate what that threshold is for PwVI, and how that threshold compares with the population more generally. There is also the need for a better understanding of what types of support and intervention are needed for PwVI, and what skills and resources are needed to enable PwVI to negotiate the constraints of future lockdowns or ongoing measures relating to distancing.

## Conclusion

Overall, the findings of this study help raise awareness of the effect the lockdown has had on PwVI, while also responding to the call for more data on the impacts of COVID-19 on people with disability (Reed et al., 2020). The findings show that participation was particularly reduced during the lockdown for PwVI that have a low household income, own a guide dog, and have underlying health problems specific to COVID-19. This emphasises the importance of support and intervention strategies targeted at those particular groups of PwVI, for instance, in terms of additional financial assistance, guide dog or cane training, mental or other health services, or early access to a COVID-19 vaccine.

In addition, the findings show that negotiation can significantly reduce the negative effect of the lockdown on participation and well-being. This emphasises the importance of support and intervention strategies that allow PwVI to adapt their daily activities to the lockdown situation to increase participation and well-being, and to mitigate against the onset and negative effects of intrapersonal constraints, for instance, with the assistance of technological solutions or awareness campaigns targeted at PwVI, service providers, or the general public.

More generally, the findings support calls for PwVI to be added to the list of clinically vulnerable populations in the event of future lockdowns, and also support calls for a more disability inclusive response to the COVID-19 crisis in general (Armitage & Nellums, 2020; Boyle et al., 2020).
